# J-shaped association between uric acid and breast cancer risk: a prospective case–control study

**DOI:** 10.1007/s00432-023-04725-y

**Published:** 2023-03-30

**Authors:** Kexin Fan, Tengfei Sun, Fuzai Yin

**Affiliations:** 1 Department of Internal Medicine, Hebei Medical University, Shijiazhuang, Hebei China; 2 Department of Pneumology, The First Hospital of Qinhuangdao, Qinhuangdao, Hebei China; 3 Department of Gastrology, The First Hospital of Qinhuangdao, Qinhuangdao, Hebei China; 4 Department of Endocrinology, The First Hospital of Qinhuangdao, Qinhuangdao, Hebei China

**Keywords:** J-shaped association, Uric acid, Breast cancer risk, Prospective case–control study, Restricted cubic spline

## Abstract

**Background/aim:**

In terms of breast cancer risk, there is no consensus on the effect of uric acid (UA) levels. The aim of our study was to clarify the link between UA and breast cancer risk in a prospective case–control study and to find the UA threshold point.

**Methods:**

We designed a case–control study with 1050 females (525 newly diagnosed breast cancer patients and 525 controls). We measured the UA levels at baseline and confirmed the incidence of breast cancer through postoperative pathology. We used binary logistic regression to study the association between breast cancer and UA. In addition, we performed restricted cubic splines to evaluate the potential nonlinear links between UA and breast cancer risk. We used threshold effect analysis to identify the UA cut-off point.

**Results:**

After adjusting for multiple confounding factors, we found that compared with the referential level (3.5–4.4 mg/dl), the odds ratio (OR) of breast cancer was 1.946 (95% CI 1.140–3.321) (*P* < 0.05) in the lowest UA level and 2.245 (95% CI 0.946–5.326) (*P* > 0.05) in the highest level. Using the restricted cubic bar diagram, we disclosed a J-shaped association between UA and breast cancer risk (*P*-nonlinear < 0.05) after adjusting for all confounders. In our study, 3.6 mg/dl was found to be the UA threshold which acted as the optimal turning point of the curve. The OR for breast cancer was 0.170 (95% CI 0.056–0.512) to the left and 1.283 (95% CI 1.074–1.532) to the right of 3.6 mg/dl UA (*P* for log likelihood ratio test < 0.05).

**Conclusion:**

We found a J-shaped association between UA and breast cancer risk. Controlling the UA level around the threshold point of 3.6 mg/dl provides a novel insight into breast cancer prevention.

## Introduction

Uric acid (UA) is the final product of purine metabolism excreted by the kidneys and gut. Elevated UA levels were closely associated with metabolic syndrome (MetS) (Bowden et al. 2022). In recent years, MetS has increasingly become a public health issue and recognized as a significant risk factor of breast cancer (Wu et al. 2018). It encompasses a group of metabolic disorders characterized by insulin resistance, central obesity, high dyslipidemia and blood pressure. However, as the key player linking MetS with inflammation and cancer, UA has been overlooked, and little research has been conducted involving the association between UA levels and breast cancer risk. High UA may promote the development of cancer through inflammatory response and oxidative stress, and urate-lowering drugs may have potential anti-cancer effects (Mi et al. 2020). There is also evidence from previous studies that UA contributes towards cancer risk, progression, and mortality (Dovell F, Boffetta P 2018; Cho et al. 2018). Notably, UA is also the most abundant antioxidant in the blood and has been shown to have some anti-cancer effects (Ames et al. 1981; Mirończuk-Chodakowska et al. 2018). Some studies have revolved about a J or U-shaped relationship between UA levels, cardiovascular disease, and mortality (Cho et al. 2018; Suliman et al. 2006). We speculated that there may also exist a nonlinear relationship between UA and breast cancer risk, which may partly explain the conflicting research results.

The report on the global Cancer Burden in 2020 shows that there are 2.26 million new breast cancer cases in women worldwide, accounting for 11.7% of the world's new cancer cases, which makes it the most common one, exceeding lung cancer. In 2020, there were approximately 420,000 new cases of BC and 117,000 deaths among Chinese women, ranking first in the world in incidence and in female cancer deaths (Cao et al. 2021). Currently, there are conflicting studies on UA and breast cancer. According to some studies, UA and breast cancer risk in women are negatively correlated (Yiu et al. 2017). However, Feng et al. studied postmenopausal women and showed that UA acted as a significant mediator in the correlation between obesity and breast cancer (Feng et al. 2021). The reason for controversial results may be the dual function of UA and different factors, such as recall bias, the various research confounding factors, and individual investigators’ subjective bias. Most of these studies assumed the existence of a linear relationship between UA and breast cancer, thus ignoring the hidden nonlinear relationship. Consequently, we initiated this prospective study aiming to further clarify the relationship between UA and breast cancer.

## Materials and methods

### Study population

We recruited the participants in our research from January 2022 to November 2022. The inclusion criteria for the cases were females: (1) all patients were newly diagnosed with invasive ductal breast cancers through postoperative pathology; (2) 20 years of age or older. The exclusion criteria were: (1) presence of other malignant tumors; (2) past surgery, chemotherapy, targeted therapy, endocrine therapy, and other therapy for any malignant tumors; (3) chronic kidney diseases; (4) consumption of any drug that regulates UA level; (5) inadequate clinical information.

The inclusion criteria for the control group were: (1) females who had completed a regular physical examination in the same hospital at the same period; (2) age matched with the breast cancer patients. The exclusion criteria were: (1) presence of other malignant tumors; (2) positive ultrasound breast scans and/or mammographic screening results; (3) cancer evidence or history; (4) chronic kidney diseases; (5) consumption of any drug that regulates UA level; (6) inadequate clinical information.

Finally, 1050 participants (525 cases and 525 controls) conforming to the above-mentioned criteria were included in the study. The Ethics Committee approved this study.

### Data collection

Medical records and comprehensive questionnaires were used to collect data by trained investigators. The questionnaire included age, sex, history of type 2 diabetes mellitus (T2DM), smoking, alcohol drinking, family history of cancer, age at menarche (years), age at first pregnancy (years) and menopause or not. Height (cm) and weight (kg) were measured for all subjects and body mass index (BMI) was also calculated. All participants underwent a physical examination.

Metabolic indicators such as low-density lipoprotein cholesterol (LDL-C, mmol/l), fasting glucose (mmol/l), triglycerides (TG, mmol/l), and UA (mg/dl) were collected through medical records. Blood tests were performed during hospitalization through standard methods using an autoanalyzer. Blood samples were taken after fasting for at least 8 h.

### Data analysis

To perform the statistical analysis, we used R software (version 4.1.0, R Foundation for Statistical Computing, Vienna, Austria). The threshold effect of UA was analyzed using the Empower Stats statistical software (http://www.empowerstats.com). The Kolmogorov–Smirnov method was used for normality testing. For continuous data with normal deviation, means and standard deviation were used. In the absence of a normal deviation, medians and interquartile ranges were calculated. Categorical data were described by frequency and percentages. Continuous variables with normal distribution and without normal distribution were compared by ANOVA and Kruskal–Wallis test, respectively. A *χ*^2^ test was applied to analyze the differences of categorical variables.

We sorted the participants into six groups ordered by increasing UA levels (mg/dl): Group A ≤ 3.5, 3.5 < Group B ≤ 4.4, 4.4 < Group C ≤ 5.4, 5.4 < Group D ≤ 6.4, 6.4 < Group E ≤ 7.4, and Group F > 7.4. Group B, which showed the lowest breast cancer risk, was treated as the reference group.

To address the nonlinear association of UA and breast cancer, multivariable logistic regression with a restricted cubic spline (RCS) functions model was conducted. In multivariable logistic regression, we adjusted for all the confounder variables, such as age, BMI, T2DM, family history of cancer, smoking, alcohol drinking, menopause, age at menarche, age at first pregnancy, LDL, fasting glucose, and TG. We used four nodes (5th, 25th, 65th, and 95th percentiles of UA) in the RCS model to disclose the relationship between UA and breast cancer risk. Then, we applied smooth curve fitting and the threshold effect analysis to obtain the optimal inflection point. We calculated multiple models using a percentile (between 10 and 90%) of UA as inflection point. To achieve the threshold effect of the UA, we established a two-piecewise linear regression model to the left and right of the inflection point. We identified the optimal inflection point according to the maximum log likelihood. To determine whether a threshold exists, we compared the one-line (non-segmented) model with the segmented regression model using the log-likelihood ratio test; *P* < 0.05 was regarded as statistically significant. The output table (Table 3) includes a two-piece-wise regression which was picked through selecting the optimal inflection point.

## Results

1. Participants’ characteristics

Using the above criteria, 1050 subjects (525 cases and 525 controls) were enrolled in our study. Participants ranged in age from 21 to 85 at enrollment. The average age of the two groups were both 56 years. All the continuous data conformed to a normal distribution. The general characteristics of the enrolled subjects are recorded in Table 1. The data are presented according to various UA levels. Age, BMI, T2DM, family history of cancer, menopause, LDL-C, and TG were different among various UA groups (*P* < 0.05). Among all the groups, T2DM incidence, family history of cancer, and TG were higher in Group F; menopause incidence and age were higher in Group D. Moreover, in Group E, BMI and LDL-C achieved the highest values.

2. Association of UA with breast cancer risk

**Table 1:** Baseline characteristics divided by UA levels

UA (mg/dl)	GroupA	Group B	GroupC	GroupD	GroupE	GroupF	*P* value
	≤ 3.5 (*n* = 144)	3.5–4.4 (*n* = 276)	4.4–5.4 (*n* = 327)	5.4–6.4 (*n* = 184)	6.4–7.4 (*n* = 69)	> 7.4 (*n* = 50)	
Age, years	51.10 ± 11.52	54.66 ± 11.42	57.33 ± 11.41	57.96 ± 10.77	57.48 ± 12.84	56.30 ± 11.65	0.018
BMI (kg/m^2^)	21.57 ± 3.00	21.90 ± 3.97	23.65 ± 4.14	23.83 ± 3.86	25.00 ± 2.65	24.34 ± 4.67	< 0.001
T2DM, %	9(6.25%)	8(2.90%)	19(5.81%)	19(10.33%)	6(8.70%)	9(18.00%)	0.001
Family history of cancer, %	9(6.25%)	12(4.35%)	17(5.20%)	20(10.87%)	4(5.80%)	6(12.00%)	0.044
Smoking, %	4(2.78%)	4(1.45%)	14(4.28%)	6(3.26%)	2(2.90%)	1(2.00%)	0.490
Alcohol consumption, %	6(4.17%)	8(2.90%)	12(3.67%)	2(1.09%)	2(2.90%)	2(4.00%)	0.607
menapause	82(56.94%)	159(57.61%)	242(74.01%)	145(78.80%)	51(73.91%)	39(78.00%)	< 0.001
Age at menarche, years	14.58 ± 1.47	14.77 ± 1.52	14,60 ± 1.49	14.89 ± 1.62	14.28 ± 1.62	14.82 ± 1.69	0.062
Age at first pregnancy, years	26.00 ± 2.39	26.00 ± 2.91	26.24 ± 3.00	26.56 ± 2.68	25.84 ± 2.70	26.18 ± 2.16	0.314
LDL-C (mmol/l)	2.48 ± 0.76	2.43 ± 0.79	2.78 ± 0.92	3.20 ± 1.06	3.29 ± 1.29	3.23 ± 0.94	< 0.001
TG (mmol/l)	1.11 ± 0.68	1.11 ± 0.57	1.34 ± 0.66	1.60 ± 0.72	1.91 ± 1.11	2.02 ± 0.97	< 0.001
Fasting glucose (mmol/l)	5.21 ± 1.13	5.39 ± 1.85	5.47 ± 1.30	5.59 ± 1.48	5.28 ± 1.45	5.88 ± 2.39	0.074

For categorical variables *n* (%) is shown. For continuous variables, mean SD is shown

*UA* uric acid, *BMI* body mass index, *T2DM* type 2 diabetes mellitus, *LDL-C* low density lipoprotein cholesterol, *TG* triglyceride

We sorted the participants into six groups according to their baseline UA levels (mg/dl): Group A ≤ 3.5 (*n* = 144), 3.5 < Group B ≤ 4.4 (*n* = 276), 4.4 < Group C ≤ 5.4 (*n* = 327), 5.4 < Group D ≤ 6.4 (*n* = 184), 6.4 < Group E ≤ 7.4 (*n* = 69), and Group F > 7.4 (*n* = 50). The OR between UA and breast cancer risk are presented in Table 2. Group B shows the lowest breast cancer risk. Thus, we treated it as a referential group. Based on model 1, compared with the reference group, the OR of the lowest level of UA (≤ 3.5 mg/dl) was 1.938 (95% CI 1.277–2.941) (*P* < 0.05) through the univariate logistic regression analysis. The OR increased to 5.963 (95% CI 2.974–11.954) in the highest level of UA (> 7.4 mg/dl) (*P* < 0.05). Regarding model 2, after adjusting for age, BMI, T2DM, family history of cancer, smoking, alcohol drinking, menopause, age at menarche, and age at first pregnancy, compared with the reference group, the OR at the lowest level of UA was 1.928 (95% CI 1.202–3.095) (*P* < 0.05), then the OR increased to 4.093 (95% CI 1.924–8.709) at the highest level of UA (*P* < 0.05). Based on the model 2, we further incorporated LDL-C, fasting glucose, and TG to model 3. The results demonstrated that, comparing with the reference group, the OR of the lowest level of UA was 1.946 (95% CI 1.140–3.321) (*P* < 0.05). The highest UA level was associated with an elevated breast cancer risk as well. However, the result was not statistically significant in Model 3 after incorporating all the confounders (OR, 2.245, 95% CI 0.946–5.326) (*P* > 0.05); details were shown in Table 2.

3. The dose relationship between UA and breast cancer

**Table 2:** Comparison of breast cancer risk among various UA groups

	Model 1		Model 2		Model 3	
	OR	*P* value	OR	*P* value	OR	*P* value
Group A (*n* = 144)	1.938 (1.277, 2.941)	0.002	1.928 (1.202, 3.095)	0.007	1.946 (1.140, 3.321)	0.015
Group B (*n* = 276)	1		1		1	
Group C (*n* = 327)	1.504 (1.078, 2.099)	0.016	1.441 (0.979, 2.123)	0.064	1.055 (0.679, 1.638)	0.812
Group D (*n* = 184)	3.338 (2.256, 4.940)	< 0.001	2.650 (1.697, 4.139)	< 0.001	1.348 (0.794, 2.286)	0.269
Group E (*n* = 69)	4.519 (2.534, 8.059)	< 0.001	4.483 (2.272, 8.845)	< 0.001	2.763 (1.229, 6.215)	0.014
Group F (*n* = 50)	5.963 (2.974, 11.954)	< 0.001	4.093 (1.924, 8.709)	< 0.001	2.245 (0.946, 5.326)	0.067

Model1, univariate logistic regression analysis; Model2, multivariate logistic regression analysis, adjusted for age, body mass index, type 2 diabetes mellitus, family history of cancer, smoking, alcohol consumption, menopause, age at menarche, age at first pregnancy;Model3, based on model 2, low density lipoprotein cholesterol, fasting glucose, and triglyceride were adjusted. The participants were classified into six groups based on their baseline UA levels (mg/dl): Group A ≤ 3.5, 3.5 < Group B ≤ 4.4, 4.4 < Group C ≤ 5.4, 5.4 < Group D ≤ 6.4, 6.4 < Group E ≤ 7.4, 7.4 < Group F. Group B, which is associated with a lower breast cancer risk, was treated as a reference group

**Fig. 1:**
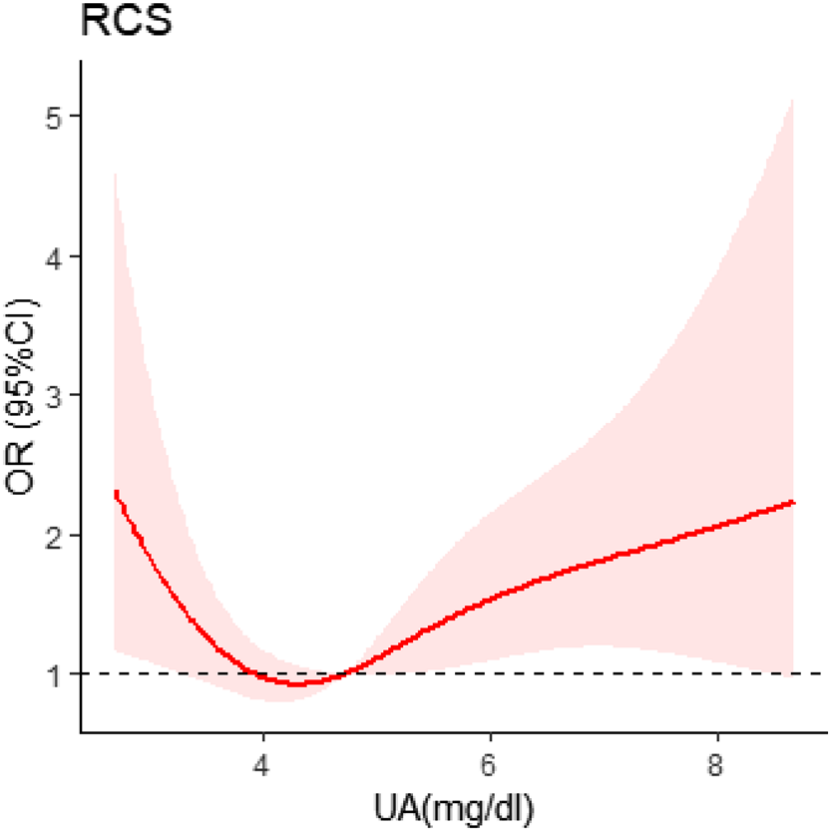
Dose–response relationship between UA levels and breast cancer risk. RCS analysis shows a J-shaped relationship between UA levels and breast cancer risk. The OR was adjusted for age, BMI, T2DM, family history of cancer, smoking, alcohol drinking, menopause, age at menarche, age at first pregnancy, LDL, fasting glucose, and TG

To evaluate the nonlinear association between UA and breast cancer risk, we applied a model which combined multivariate logistic regression analysis with RCS. The RCS displayed an apparent decrease of the breast cancer risk within the lower levels of UA. The lowest risk was achieved at a UA concentration around 3.5–4.5 mg/dl and increased thereafter. Thus, the fully adjusted RCS showed a strong J-shaped association between UA blood levels and breast cancer risk (Fig. 1) (*P* for nonlinearity < 0.05).

In addition, we tested the UA threshold effects on breast cancer risk. We established a smooth curve fitting through logistic regression analysis. The RCS and the smooth curve fitting confirmed the existence of a linear relationship between UA and breast cancer. We conducted a two-piecewise logistic regression model and we analyzed every possible model within the range 10–90% of UA as turning point. We selected the model with the optimal inflection point and we conducted a piecewise linear regression model on both the lower and higher levels of the optimal inflection point, which provided maximum log likelihood. Using this method, we found that the optimal inflection point of UA level was 3.6 mg/dl. The OR for breast cancer was 0.170 (95% CI 0.056–0.512) and 1.283 (95% CI 1.074–1.532) to the left and right of 3.6 mg/dl, respectively (*P* for log likelihood ratio < 0.05). These results demonstrated that the link between UA and breast cancer risk can be satisfactorily explained by a two-piecewise model, and the threshold was regarded as statistically significant, as shown in Table 3.

**Table 3:** Threshold effect analysis of UA on breast cancer risk

UA (mg/dl)	Model 1		Model 2		Model 3	
	OR (95%CI)	*P* value	OR (95%CI)	*P* value	OR (95%CI)	*P* value
Inflection point	3.6		3.6		3.6	
< Inflection point	0.233 (0.112, 0.485)	0.001	0.216 (0.086,0.542)	< 0.001	0.170 (0.056,0.512)	0.002
> Inflection point	1.607 (1.421, 1.817)	< 0.001	1.500 (1.284, 1.751)	< 0.001	1.283 (1.074, 1.532)	0.006
*P* for log likelihood ratio test	< 0.001		< 0.001		< 0.001	

UA, uric acid. Model 1, univariate logistic regression analysis; Model 2, multivariate logistic regression analysis, adjusted for age, body mass index, type 2 diabetes mellitus, family history of cancer, smoking, alcohol consumption, menopause, age at menarche, age at first pregnancy; Model 3, based on model 2, low density lipoprotein cholesterol, fasting glucose and triglyceride were adjusted

## Discussion

Metabolic risk factors of breast cancer tend to be less noticeable than traditional risk factors such as fertility and family history. It has been gradually recognized that MetS is a detrimental factor closely related to many cancers such as breast, colorectal, and prostate cancers (Akinyemiju et al. 2022; López-Jiménez et al. 2022; Hernández-Pérez et al. 2022; Dong et al. 2021). Although UA level is not a defined component of MetS, there are close links between them. UA has been implicated in the onset of MetS, and reducing UA levels can prevent or reverse it (King et al. 2018; Reungjui et al. 2007; Son et al. 2022). Uric acid is a pro-inflammatory entity that promotes the occurrence and prognosis of cardiovascular disease and cancer. Anti-uric acid drugs have shown potential therapeutic effects on cancer (Mi et al. 2020). Moreover, as a natural antioxidant, UA exhibits a robust (up to 60%) free radical scavenging activity in human blood (Ames et al. 1981; Mirończuk-Chodakowska et al. 2018). However, the association of UA with breast cancer has not received sufficient attention. Owing to inadequate understanding of the association of UA and breast cancer, the limited research on the subject has yielded contradictory results (Yiu et al. 2017; Feng et al. 2021; Kühn et al. 2017; Yan et al. 2015). Thus, we tried to display the association between UA and breast cancer through a prospective study to evaluate the links between them.

In this study, we found that both low and high UA levels coincided with an elevated risk of breast cancer. Previous analyses of UA and cancer risk yielded heterogeneous results. A prospective cohort study including 19,518 Chinese postmenopausal women revealed links between BMI and UA with breast cancer risk. Furthermore, this study revealed that UA was a significant mediator in obesity-related breast cancer (Feng et al. 2021). There was also evidence that UA was an independent risk factor of breast cancer (Hong et al. 2022). However, some studies have shown the opposite result. The researchers hypothesized that UA acted as an antioxidant defense against cancer (Ames et al. 1981; Mirończuk-Chodakowska et al. 2018). Previous studies provide some evidence to support this hypothesis. Yiu et al. revealed that high UA levels played a protective role for several cancers, including breast cancer in females (Yiu et al. 2017).In a cohort study, it was found that low UA levels were linked with lung cancer (Horsfall et al. 2014). Additionally, UA was proven to be a protective factor for breast cancer risk and mortality, suggesting that UA acted as a favorable factor for impeding breast cancer development (Kühn et al. 2017). Our study also showed that the lowest UA levels corresponded to the highest risk of breast cancer. The dual role of UA in cancer may partly explain these differences. The study-specific confounding factors such as region, ethnicity, diet, comorbidities, and bias of individual investigators in the selection of populations and measures may explain the observed differences. In addition, the previous studies did not consider the nonlinear relationship between UA and breast cancer risk.

Our study showed an apparent reduction of breast cancer risk within the lower levels of UA. The lowest risk was reached around 3.5–4.5 mg/dl and increased thereafter with a relatively moderate trend. The lowest UA levels showed a 1.946-fold increase in breast cancer risk compared with the reference group (*P* < 0.05). The highest UA levels corresponded with a 2.245-fold increase in breast cancer risk, although it was not statistically significant (*P* > 0.05). This may be explained by the dual role of UA against breast cancer. Notably, the association between UA and breast cancer risk presented a J-shaped trend, even after adjusting baseline characteristics, other common risks, and metabolic factors (*P*-nonlinear < 0.05). The inflection point in the curve occurred at 3.6 mg/dl. Indeed, the nonlinear association presented as a U-shaped or J-shaped curve has also been found between UA levels and mortality through several cohort studies (Cho et al. 2018; Suliman et al. 2006; Kuo et al. 2013; Mazza et al. 2007). Huang CF also reported that high UA levels were a risk factor for pancreatic cancer in women and gallbladder cancer in men. Furthermore, the study identified a U-shaped association between UA and liver cancer risk (Huang et al. 2020). Based on data from the UK Biobank, Mi N et al. showed that UA and colon cancer risk is also characterized by a U-shaped association (Mi et al. 2022). A nonlinear association between UA and mortality was also found by Hu L et al. They indicated that the inflection point of UA was 4 mg/dl and 6 mg/dl for females and males, respectively (Hu et al. 2020). It is worth noting that the inflection point in females is close to the threshold value found in our study.

Although the mechanisms by which UA is linked with breast cancer has remained elusive, increasing evidence has emerged to support the possible role of UA ability in carcinogenesis. The activated inflammatory pathway deriving from UA and the outcome of elevated reactive oxygen species (ROS) are involved in the interplay of UA and the immunity system (Mi et al. 2020). An oxidative stress emerges when ROS production prevails over natural organism antioxidants (Zhou et al. 2022). Oxidative stress and the corresponding oxidative damage significantly contribute to cancer process (Klaunig 2018). Cancer environment promotes ROS production owing to oxidative stress (Zhou et al. 2022). Epidemiological studies have associated chronic oxidative stress with cancer as well (Islam et al. 2019). Furthermore, UA increases inflammatory stress, which plays an important role in cancer pathogenesis (Joosten et al. 2020). Chronic inflammation in the tumor microenvironment embraces well-known influences on cancer progress and immunity. The mammalian target of rapamycin (mTOR) pathway activation linked the initiating inflammatory pathways with neoplastic transformation. In most studies, UA induced the AKT/mTOR pathway, elevating all assimilating pathways, containing protein, lipid, and nucleotide synthesis, while obstructing autophagy, leading to cell proliferation (Allegrini et al. 2022). Meanwhile, UA acted as a strong antioxidant, contributing to most of the antioxidant capability of plasma (Ames et al. 1981; Mirończuk-Chodakowska et al. 2018; Oliveira and Burini 2012). As an antioxidant, UA exhibits a strong protective effect against cancer, in part by inhibiting oxygen radical production and lipid peroxidation (Ames et al. 1981; Mirończuk-Chodakowska et al. 2018; Lin et al. 2021). Moreover, UA deficiency is intimately linked with poor nutritional status (Park et al. 2017). Malnourishment is always correlated with inadequate intake of vitamin D (Merker et al. 2019). Lack of antioxidants and vitamin D always coexisted with an elevated risk of breast cancer (Francis et al. 2021). Our study shows that the lowest UA level coincided with increased breast cancer risk.

To the best of our knowledge, our study is the first to reveal the potential nonlinear J-shaped association between UA and breast cancer risk and, which may also explain the conflicting findings of previous studies. Moreover, we found that the threshold point for the J-shaped breast cancer risk curve was at 3.6 mg/dl UA level. Therefore, especially for those with high risk such as family history, controlling UA around a reasonable level of 3.6 mg/dl rather than blindly reducing UA level is a significant recommendation for the prevention of breast cancer.

It should be noted, however, that there are some limitations in our study. Firstly, since this is cross-sectional research, we cannot identify the causality between UA and breast cancer. Secondly, owing to the limited sample size, it is not possible to implement stratified analyzes. Thirdly, we did not record the dietary habits linked with UA. Therefore, larger prospective studies are still needed to make up for the above limitations.

## Conclusion

In summary, our study demonstrates for the first time that there is a J-shaped association between UA and breast cancer risk. Furthermore, we determined an optimal threshold of 3.6 mg/dl UA, which acted as a trend turning point. The strong J-shaped association between UA and breast cancer provides a novel insight into breast cancer prevention. Therefore, UA levels should always be confirmed to achieve a reasonable range around 3.6 mg/dl. For a progressive defense against BC, a larger prospective study is required to ascertain the accuracy and threshold of the association for predicting breast cancer risk and elucidate the pathogenic mechanisms and explore the value of conventional and novel drugs that have been used merely as UA modulators.

## Data Availability

The data that support the findings of this study are available upon reasonable request from the corresponding author.
